# Down but Not Out: The Role of MicroRNAs in Hibernating Bats

**DOI:** 10.1371/journal.pone.0135064

**Published:** 2015-08-05

**Authors:** Lihong Yuan, Fritz Geiser, Benfu Lin, Haibo Sun, Jinping Chen, Shuyi Zhang

**Affiliations:** 1 Guangdong Entomological Institute, Guangzhou, China; 2 Guangdong Public Laboratory of Wild Animal Conservation and Utilization, Guangzhou, China; 3 Guangdong Key Laboratory of Integrated Pest Management in Agriculture, Guangzhou, China; 4 Center for Behavioural and Physiological Ecology, Zoology, University of New England, Armidale, Australia; 5 Animal Husbandry and Veterinary Bureau of Huadu District, Guangzhou, China; 6 MininGene Biotechnology Co. Ltd, Beijing, China; 7 Institute of Molecular Ecology and Evolution, Institutes for Advanced Interdisciplinary Research, East China Normal University, Shanghai, China; Kunming University of Science and Technology, CHINA

## Abstract

MicroRNAs (miRNAs) regulate many physiological processes through post-transcriptional control of gene expression and are a major part of the small noncoding RNAs (snRNA). As hibernators can survive at low body temperatures (T_b_) for many months without suffering tissue damage, understanding the mechanisms that enable them to do so are of medical interest. Because the brain integrates peripheral physiology and white adipose tissue (WAT) is the primary energy source during hibernation, we hypothesized that both of these organs play a crucial role in hibernation, and thus, their activity would be relatively increased during hibernation. We carried out the first genomic analysis of small RNAs, specifically miRNAs, in the brain and WAT of a hibernating bat (*Myotis ricketti*) by comparing deeply torpid with euthermic individual bats using high-throughput sequencing (Solexa) and qPCR validation of expression levels. A total of 196 miRNAs (including 77 novel bat-specific miRNAs) were identified, and of these, 49 miRNAs showed significant differences in expression during hibernation, including 33 in the brain and 25 in WAT (*P*≤0.01 &│logFC│≥1). Stem-loop qPCR confirmed the miRNA expression patterns identified by Solexa sequencing. Moreover, 31 miRNAs showed tissue- or state-specific expression, and six miRNAs with counts >100 were specifically expressed in the brain. Putative target gene prediction combined with KEGG pathway and GO annotation showed that many essential processes of both organs are significantly correlated with differentially expressed miRNAs during bat hibernation. This is especially evident with down-regulated miRNAs, indicating that many physiological pathways are altered during hibernation. Thus, our novel findings of miRNAs and Interspersed Elements in a hibernating bat suggest that brain and WAT are active with respect to the miRNA expression activity during hibernation.

## Introduction

Hibernation (multiday torpor) and daily torpor in heterothermic mammals are characterized by substantial, temporal reductions of energy expenditure and body temperature (T_b_). Torpor is often expressed when animals are exposed to low ambient temperature (T_a_), and the decrease in energy use is crucial for survival during adverse conditions and food shortages [1]. Mammalian hibernation is an extreme example of hypometabolism with metabolic rate depression often to <5% of the euthermic rate, a minimum T_b_ of approximately 0°C, and cell preservation during prolonged dormancy periods [2, 3]. The physiological changes, such as the decrease and subsequent increase of metabolism and T_b_ during entry and arousal from torpor, are active and adaptive processes. They are often initiated in response to changes in environmental temperature and photoperiod and require the coordination of core and peripheral tissues by the brain [4, 5]. Importantly, the carbohydrate oxidation pathway shifts upon hormone activation towards lipid metabolism, and fat (mainly stored in white adipose tissue, WAT) becomes the primary energy source during the hibernation season [6]. Considering the brain’s important role in integrating peripheral tissues and WAT to provide energy during hibernation, we hypothesized that to adequately perform these tasks, both must be activated during hibernation, in contrast to other tissues and organs, where metabolism is generally suppressed [7].

A detailed understanding of the molecular mechanisms of this phenomenon would improve our understanding of hibernation at the subcellular level, but may also be useful in human medicine for improving organ transplantation, preventing skeletal muscle atrophy during long-term bed rest, and suppressing carcinogenesis [7]. In the past ten years, molecules that regulate hibernation and the molecular mechanisms underlying hibernation have been identified by transcriptome analysis [8–12]. Thousands of genes and proteins are differentially expressed in many species during hibernation [12–17]. However, the precise and timely control of gene expression during hibernation remains unclear.

Previous studies indicated that post-transcriptional regulation occurs during hibernation [18]. MicroRNAs (miRNAs) are short RNA molecules (18–25nt), and they are known to play key roles in directing post-transcriptional activity of genes involved in all cellular processes, including differentiation and development [19]. Since Morin and colleagues [20] first proved that miRNAs regulate ground squirrel [*Ictidomys* (*Spermophilus) tridecemlineatus*] hibernation, many detailed studies have analyzed miRNAs during torpor [18, 21–25] and found that miRNAs are involved in cold adaptation, epigenetic control and metabolic rate depression [26–28]. Differential miRNA expression, and thus their target genes, is an important component of the molecular adaptation required by organisms to respond to environmental changes, especially heterothermic mammals [18, 26–28].

There are more than 1100 species of bats (order Chiroptera), and they are among the most geographically widespread mammals [29]. Most bat families include species that enter some form of torpor, whereas multiday torpor (i.e., hibernation) occurs in at least five families [30]. The wide taxonomic diversity of heterothermy in bats suggests that bats are a good model to explore the evolutionary history of mammalian heterothermy [31]. Importantly, unlike ground squirrels, for which most molecular work on hibernation has been conducted, bats are not strongly seasonal in their torpor expression and many use multiday torpor throughout the year [32]. Therefore, a systematic study of genes or proteins involved in bat hibernation by comparing active and hibernating individuals will help us better understand the molecular mechanisms of hibernation and to explore the evolution of mammalian heterothermy [11, 31, 33, 34]. However, a systematic analysis of small RNAs, especially miRNAs, with regard to function and evolution of torpor in bats is currently lacking.

The latest 7× coverage genome sequence of *Myotis lucifugus* (http://www.ensembl.org/Myotis_lucifugus/Info/Index) provides us with the opportunity to survey miRNA expression in hibernating bats and to conduct comparative analyses with sequences from other metazoan species. Moreover, the RNA-seq sequencing-by-synthesis technique allows to sequence millions of short cDNAs and increases sensitivity by reducing personal error in library construction [35]. Based on the Illumina/Solexa sequencing platform and combined with qPCR validation, we performed the first in-depth miRNA analysis on two key tissues (brain and WAT) of the hibernating bat *Myotis ricketti*, which is congeneric with *M*. *lucifugus*. Our study aimed to discover bat-specific miRNAs, illustrate their potential role during bat hibernation, and contribute to the understanding of the evolution of mammalian heterothermy.

## Methods

### Ethics statement

All procedures involving animals were performed by strictly following the Guidelines and Regulations for the Administration of Laboratory Animals (Decree No. 2 of the State Science and Technology Commission of the People’s Republic of China on November 14, 1988) and were approved by the Animal Ethics Committee of East China Normal University (20080729).

### Sample and RNA preparation

A total of six *Myotis ricketti* individuals with a body mass of 18–22 g were used in this study. Three active *M*. *ricketti* bats (T_b_~36°C) were captured using mist nets in the Fangshan area (Beijing, China; 39°48′N, 115°42′E) on October 26, 2008 (T_a_ = 21°C). On March 6, 2009, three bats in deep hibernation (T_b_~9°C) were captured in the same location (T_a_ = 7°C). The field studies did not involve endangered or protected species, and no specific permissions were required for *M*. *ricketti* in the Fangshan area. The bats were sacrificed humanely by decapitation immediately after measuring T_b_ and body mass. Whole brain and white intra-abdominal adipose tissue (WAT) of hibernating and euthermic bats were rapidly removed, immediately frozen in liquid nitrogen, and stored at -80°C until RNA extraction.

Total RNA was isolated from entire brains and adipose tissues (~0.2 g) of six bats using the RNAiso kit (TakaRa, Japan) according to the manufacturer’s instructions, and the concentration and RNA Integrity Number (RIN) was determined by an Agilent 2100 bioanalyzer. RNA samples were pooled to construct the transcriptome sequencing library as described previously [12, 21]. Briefly, total RNA from bat brains of the same stage were mixed in equal amounts (10 μg each) into two pooled samples, representing the hibernation and active state, hereafter referred to as HB (hibernating brain) and AB (active brain). Similarly, total WAT RNA from hibernating and active bats (10 μg each) were pooled and referred to as HA (hibernating WAT), or AA (active WAT). In total, four RNA pools (two tissues in two physiological states) were used to construct small RNA libraries. The RNA quality and quantity were as follows: HB: 480 ng/μl, RIN = 8.3, 28S/18S = 1.2; AB: 2210 ng/μl, RIN = 8.1, 28S/18S = 1.5; HA: 390 ng/μl, RIN = 8.4, 28S/18S = 1.4; AA: 1440 ng/μl, RIN = 7.5, 28S/18S = 1.5.

### Construction and high-throughput sequencing of small RNA libraries

Small RNA cDNA libraries were constructed as described previously [36]. Briefly, small RNA (18~33 nt) was purified and enriched from each total RNA sample by polyacrylamide gel electrophoresis (PAGE), and 10 μg small RNA was ligated with the proprietary adapters used for cDNA synthesis and library construction, then Solexa sequencing-by-synthesis was performed (Illumina). All small RNA data series were submitted to NCBI Gene Expression Omnibus (GEO) with the accession number GSE29053.

### Filter of small RNA reads

Individual sequence reads with base quality scores were produced by Illumina/Solexa. After removing contaminant reads (adapters, low quality and redundant reads), clean unique reads were mapped onto the *Myotis lucifugus* genome (http://asia.ensembl.org/Myotis_lucifugus/Info/Index) using the Bowtie program [37]. Perfectly mapped reads were scanned against the metazoan mature miRNA in the Sanger miRBase (Release 19) [38] to identify orthologs of known miRNAs using the Patscan program [39] with two mismatches allowed. Non-conserved unique reads were screened against Rfam databases (Release 10) [40] to filter the sequences of tRNA, rRNA, snoRNA, and other ncRNAs except miRNAs using Bowtie. Reads that matched to the genome more than 20 times were removed by miRDeep [41], and reads sequenced only one time were also removed. Finally, the remaining reads were considered potential miRNA reads and were used for miRNA identification.

### MiRNA identification

MiRNA and its antisense strand can both be sequenced from the same precursor. Thus, to avoid repeated predictions and to reduce calculations, candidate reads whose distance in the reference genome were <200 nt were combined and examined as one genomic block. For each block, 150 nt of upstream and downstream extensions were extracted for secondary structure prediction. First, inverted repeats (IR) with stem-loop or hairpin structures were identified by Einverted of Emboss [42], with the following parameters: threshold = 30, match score = 3, mismatch score = 3, gap penalty = 6, and maximum repeat length = 240, as described previously [43]. The IR secondary structure was then predicted by RNAfold [44] with 10 nt upstream and downstream extensions and evaluated by MirCheck [43].

Predicted precursors that miRNA and antisense strand can be found in both arms were deemed miRNA candidates. Moreover, if the candidates have two or more unique reads resulted from the ±2 nt bias during cleavage and located at mature positions, they were deemed highly probable. When several length variants of the same miRNA were sequenced, only variants with the highest representation were considered. Finally, the miRNA candidates were submitted again to miRBase, and the miRNA precursors (hairpins) that passed MirCheck were manually inspected against the canonical miRNA structure to remove false predictions.

### Repeat-derived siRNA detection

To screen repeat-derived small interfering RNA (siRNAs), the repeat sequences of the *M*. *lucifugus* genome were annotated by Repeatmasker (http://www.repeatmasker.org/) [45]. Reads that perfectly matched the *M*. *lucifugus* genome were aligned to repetitive elements using Bowtie, and reads that perfectly matched the repeats were considered genomic repeats-derived siRNAs.

### Differential miRNA expression analysis and target prediction

Differentially expressed miRNAs that were statistically significant in relative abundance (reflected by Transcript Per Million, TPM) between hibernating and active states were identified by the edgeR function in Bioconductor [46]. Empirical Bayes estimation and exact tests based on the negative binomial distribution were used, and *P*≤0.01 & │logFC│≥1 considered statistically significant.

As there is no or little information about 3’ untranslated region (UTR) of *M*. *lucifugus* reference genes in database of Ensembl (http://www.ensembl.org/Myotis_lucifugus/Info/Index), to predict miRNA target genes, we first identified the orthologs of *M*. *lucifugus* genes by searching against *Homo sapiens* mRNA (http://www.ncbi.nlm.nih.gov/RefSeq/) with tblastx (e value ≤1e-10 and identity ≥60%) [47]. Orthologs with the best hits were kept. The *H*. *sapiens* mRNAs were used to analyze the 3’ UTR lengths, and 93.6% (67,594/72,204) of human mRNAs had a 3’ UTR less than 3 Kb (see S1 Fig), excluding 32,559 mRNAs that did not have a 3’ UTR. Thus, the 3 Kb region downstream of *M*. *lucifugus* reference gene coding sequences were extracted and aligned with the 3’UTR of *H*. *sapiens* mRNA orthologs by CLUSTALW [48] to trim the candidate sequences of 3’ UTR of *M*. *lucifugus*. For *M*. *lucifugus* reference genes that did not have a human ortholog, the 3 Kb region downstream of the coding sequence was considered the 3’UTR. The miRNA target was then predicted using the PITA program based on the interaction between miRNAs and their targets with the default criterion and ΔΔG≤−10 kcal/mol [49]. Finally, target sequence Gene Ontology (GO) annotation was performed by Interproscan [50] and target sequences were compared to the Kyoto Encyclopedia of Genes and Genomes database (KEGG, release 50) by BLASTX at E values ≤1e-10 [47, 51]. A Perl script was used to retrieve the KO (KEGG Orthology) information from BLAST results and to establish pathway associations between target genes and databases.

### Quantitative miRNA expression by qPCR assay

Stem-loop reverse transcription (RT) qPCR was performed to quantify the expression level of 10 differentially expressed miRNAs obtained by Solexa sequencing in brains and WAT as described previously [52], and 5S rRNA was used as an endogenous control. Primers used in this study are listed in S1 Table. Each RT reaction contained 1 μg total RNA, 1× Reaction Buffer, dNTPs (5 mM each), 50 nM miRNA-specific stem-loop RT primer, 100 U RevertAid Premium Reverse Transcriptase and 0.5 U RiboLock RNase Inhibitor. RT reactions were incubated at 37°C for 30 min, 42°C for 60 min and 85°C for 5 min. PCR reactions were performed in a total of 20 μl, including 0.8 μl RT product, 10 μl 2× SYBR Green I Master Mix, 6 μM each of forward primer and reverse primer with the following program: 95°C for 10 min, followed by 40 cycles of 95°C for 15 s, 60°C for 30 s and 72°C for 15 s, then 95°C for 15 s, 60°C for 1 min and a final hold at 4°C. All reactions were performed on three biological replicates in each tissue and each physiological state, and each was run three times. Negative controls containing all reagents except template were included on each reaction plate. The 2^−ΔΔCT^ method was used to calculate relative expression (fold change); and data were analyzed by One-Way ANOVA using SPSS 18.0 software.

## Results

### Small RNA sequencing and statistics

We isolated total RNA from hibernating and active *Myotis ricketti* brain and intra-abdominal adipose tissue, and the RNA concentration and RNA Integrity Number (RIN) met the requirements for Solexa small RNA library construction and sequencing.

After small RNAs were isolated and processed for deep sequencing on the Illumina/Solexa platform, we sequenced a total of 1.56×10^7^, 1.54×10^7^, 1.53×10^7^ and 1.55×10^7^ reads from the HB, AB, HA and AA libraries, respectively. We obtained a total of 4.51×10^7^ high-quality small RNA reads after removing ambiguous reads. In these high quality reads, 6.3×10^5^ (HB), 4.2×10^5^ (AB), 1.2×10^6^ (HA), 5.4×10^5^ (AA) and 2.3×10^6^ (in total) were clean unique reads, and the percentage of small RNA reads (>18 bp) in four libraries were above 60% (see S2 and S3 Tables). The redundancies of the four libraries were 94.82% (HB), 95.85% (AB), 89.92% (HA) and 95.31% (AA), and among these, the redundancy of co-expressed unique reads among libraries was 11.3% and that of state/tissue-specific reads was 88.7% (S3 Table).

**Fig 1:**
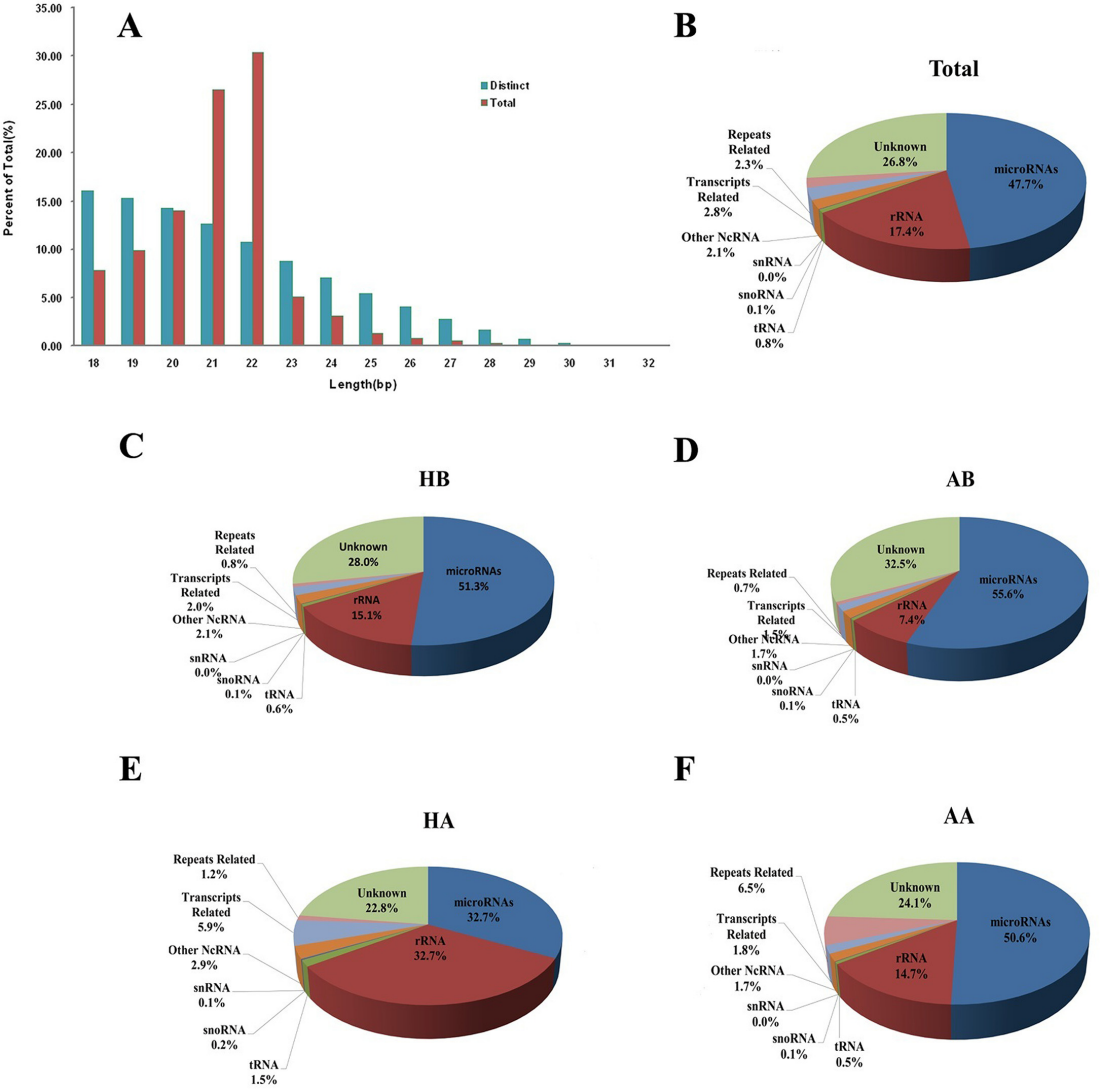
Overview of small RNA gene expression in four bat libraries generated by Solexa deep sequencing. (A) Length distribution of perfectly matched small RNA reads. The percentage of total or distinct (unique) small RNA reads that perfectly matched the reference genome are shown. (B-F) Breakdown of the proportions (in percentage) of various classes of small RNAs detected by sequencing of total/all combined, brain (HB, AB), and WAT (HA, AA). Various classes of small RNAs are shown by percentages. The miRNA family comprises the majority of small RNAs (47.7% in total). snoRNA, small nucleolar RNA; rRNA, ribosomal RNA; tRNA, transfer RNA; unknown, derived from unannotated/intergenic regions.

**Fig 2:**
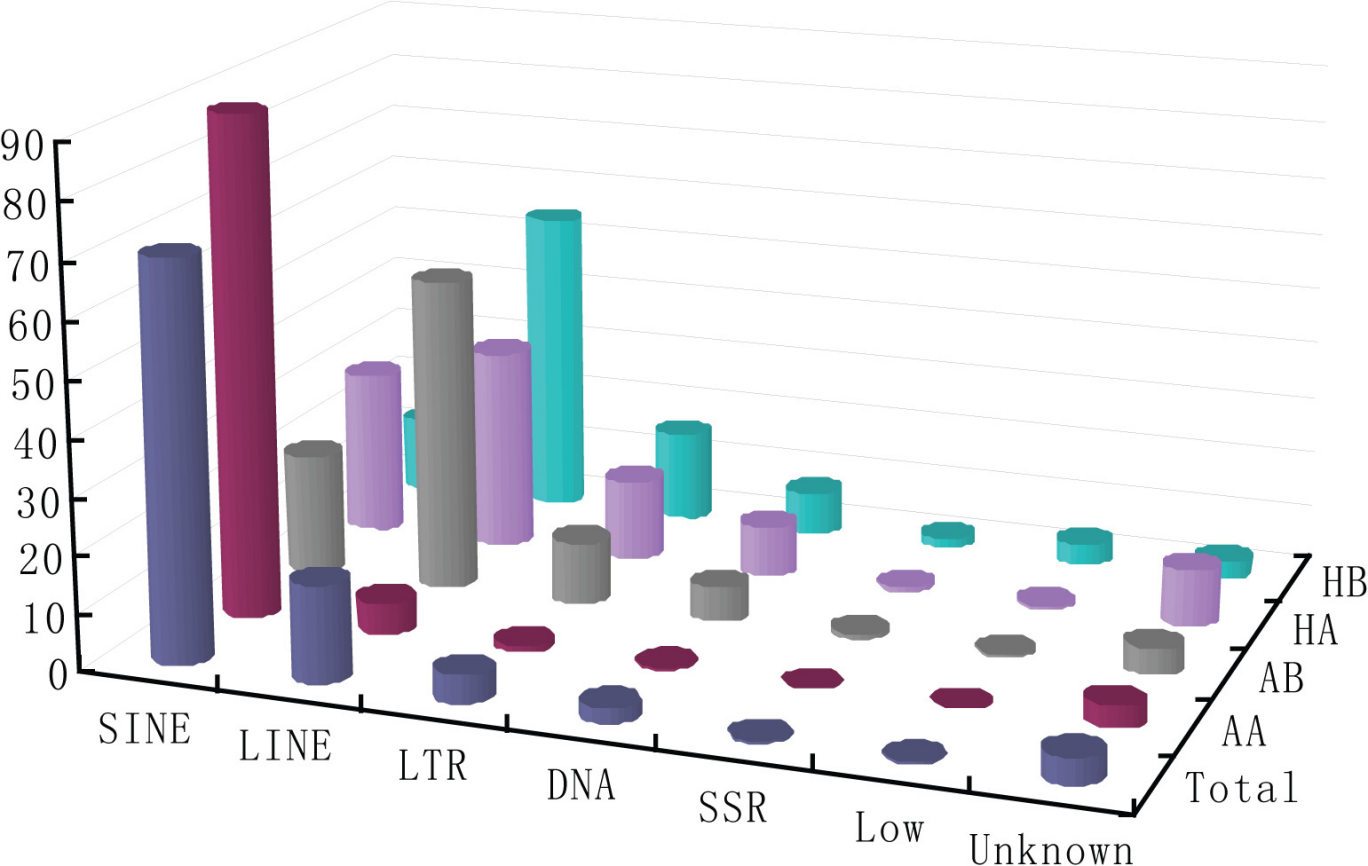
Illustration of genomic repeat-derived reads in four libraries. LINE, Long INterspersed Elements; SINE, Short INterspersed Elements; LTR, Transposable elements with Long Terminal Repeats; DNA, DNA transposons; SSR, Simple Sequence Repeats; Low, Low Complexity Sequences; unknown, derived from unannotated/intergenic regions.

Less than 5% of total small RNA reads from the bat libraries perfectly matched the *Myotis lucifugus* genome >20 times (S4 Table); thus, we removed these unique reads to avoid self-contradiction of miRNA prediction. Therefore, a total of 3.16×10^7^ remained after mapping the sequences to the *M*. *lucifugus* genome (Table 1 and S2 Fig). The size distribution of perfectly matched small RNA reads is shown in Fig 1A. We identified a total of 2.47×10^7^ potential miRNA reads, including 159,807 distinct reads, by searching against miRBase and filtering the ncRNAs and low-expressing reads (S5 Table). Subsequently, 1.51×10^7^ (47.7% in total) miRNA reads met the fold-back structure (hairpin) and MirCheck criteria, and miRNA reads were the most abundant fraction (range from 32.7% in HA to 55.6% in AB, Table 1 and Fig 1B). Other small RNAs, such as ncRNAs, genomic repeats, transcript repeats, and unknown genomic regions, comprised 20.4%, 2.3%, 2.8% and 26.8%, respectively (Table 1 and Fig 1B). Upon further inspection of genomic repeat-derived siRNAs, we determined that Short INterspersed Element (SINE) and Long INterspersed Element (LINE) were the major contributors to the four libraries (Table 1 and Fig 2).

**Table 1:** Classification of unique small RNA reads.

Classification	Total				HB				AB				HA				AA			
	Number of unique reads	Percentage (%)	Total sequences	Percentage (%)	Number of unique reads	Percentage (%)	Total sequences	Percentage (%)	Number of unique reads	Percentage (%)	Total sequences	Percentage (%)	Number of unique reads	Percentage (%)	Total sequences	Percentage (%)	Number of unique reads	Percentage (%)	Total sequences	Percentage (%)
Total Unique small RNA Reads	2323170	100	45161406	100	629751	100	12160105	100	415452	100	10012888	100	1164049	100	11549959	100	536995	100	11438454	100
Total Perfect Matched small RNA reads	720862	31.0	31590053	69.9	201875	32.1	8682951	71.4	120695	29.1	7266543	72.6	373224	32.1	7502137	65.0	152674	28.4	8138422	71.1
miRNAs^a^	7530	1.0	15068478	47.7	3365	1.7	4451386	51.3	2684	2.2	4041090	55.6	3967	1.1	2454320	32.7	3164	2.1	4121682	50.6
Non-Coding RNA^b^	71131	9.9	6443708	20.4	39956	19.8	1556064	17.9	27417	22.7	709223	9.8	47132	12.6	2798792	37.3	29197	19.1	1379629	17.0
rRNA	39273	5.4	5495818	17.4	25585	12.7	1311512	15.1	18091	15.0	539528	7.4	31298	8.4	2452251	32.7	20826	13.6	1192527	14.7
tRNA	18189	2.5	244828	0.8	7063	3.5	54006	0.6	3977	3.3	36283	0.5	7954	2.1	112091	1.5	3540	2.3	42448	0.5
snoRNA	2360	0.3	36480	0.1	1030	0.5	9531	0.1	660	0.5	5882	0.1	1280	0.3	13861	0.2	729	0.5	7206	0.1
snRNA	454	0.1	7249	0.0	214	0.1	1107	0.0	130	0.1	614	0.0	309	0.1	4669	0.1	107	0.1	859	0.0
other ncRNA	10855	1.5	659333	2.1	6064	3.0	179908	2.1	4559	3.8	126916	1.7	6291	1.7	215920	2.9	3995	2.6	136589	1.7
Transcript-derived reads	289993	40.2	873840	2.8	52157	25.8	173709	2.0	31598	26.2	105720	1.5	173270	46.4	445779	5.9	52878	34.6	148632	1.8
Genomic repeat-derived reads	67509	9.4	732080	2.3	24669	12.2	66751	0.8	11222	9.3	47251	0.7	25197	6.8	91641	1.2	10348	6.8	526437	6.5
LINE	33783	50.0	124881	17.1	12312	49.9	36423	54.6	5312	47.3	26188	55.4	13129	52.1	32850	35.8	4622	44.7	29420	5.6
SINE	5681	8.4	512264	70.0	1830	7.4	8885	13.3	941	8.4	10160	21.5	2178	8.6	26692	29.1	1016	9.8	466527	88.6
LTR	10571	15.7	35643	4.9	3637	14.7	10730	16.1	1797	16.0	5286	11.2	4007	15.9	13374	14.6	1751	16.9	6253	1.2
DNA	8644	12.8	19283	2.6	2881	11.7	5062	7.6	1674	14.9	2877	6.1	3398	13.5	8261	9.0	1444	14.0	3083	0.6
SSR	2293	3.4	3002	0.4	1012	4.1	1216	1.8	372	3.3	427	0.9	658	2.6	868	0.9	316	3.1	491	0.1
Low	2524	3.7	3194	0.4	1895	7.7	2287	3.4	178	1.6	196	0.4	338	1.3	483	0.5	173	1.7	228	0.0
Unknown Repeats	4013	5.9	33813	4.6	1102	4.5	2148	3.2	948	8.4	2117	4.5	1489	5.9	9113	9.9	1026	9.9	20435	3.9
Unknown	284699	39.5	8471947	26.8	81728	40.5	2435041	28.0	47774	39.6	2363259	32.5	123658	33.1	1711605	22.8	57087	37.4	1962042	24.1

HB: hibernating state brain; AB: active state brain; HA: hibernating state adipose tissue; AA: active state adipose tissue. rRNA, ribosomal RNA; tRNA, transfer RNA; snoRNA, small nucleolar RNA; snRNA, small nuclear RNA; LINE, Long INterspersed Elements; SINE, Short INterspersed Elements; LTR, Transposable elements with Long Terminal Repeats; DNA, DNA transposons; SSR, Simple Sequence Repeats; Low, Low Complexity Sequences; unknown, derived from unannotated/intergenic regions.

^a^ miRNA reads identified by miCheck, searched against the Sanger miRBase (Release 19) and manually inspected for canonical structure from 24656421 total potential reads (S2 Fig and S5 Table).

^b^ Screening against non-coding RNA databases (Release 10) to filter ncRNAs (tRNA, rRNA, snoRNA and other ncRNA) by Bowtie.

### MiRNA identification

To better understand the length distribution of metazoan miRNA precursors, we analyzed known miRNA precursors in five out-grouped organisms (two vertebrates and three invertebrates, S3 Fig) in miRBase. The majority of pre-mature miRNAs from these five organisms were less than 150 nt with a mean length of approximately 90 nt (S3 Fig). We mapped the potential miRNA reads onto the reference genome and retrieved 509,206 block sequences with 150 nt upstream and downstream extensions; we then used these sequences for secondary structure prediction. From these, we used Einverted of Emboss [42] and identified 380,605 potential reads with inverted repeats. We identified 90 conserved hairpins in 28 families and 101 novel hairpins (S6 Table).

**Fig 3:**
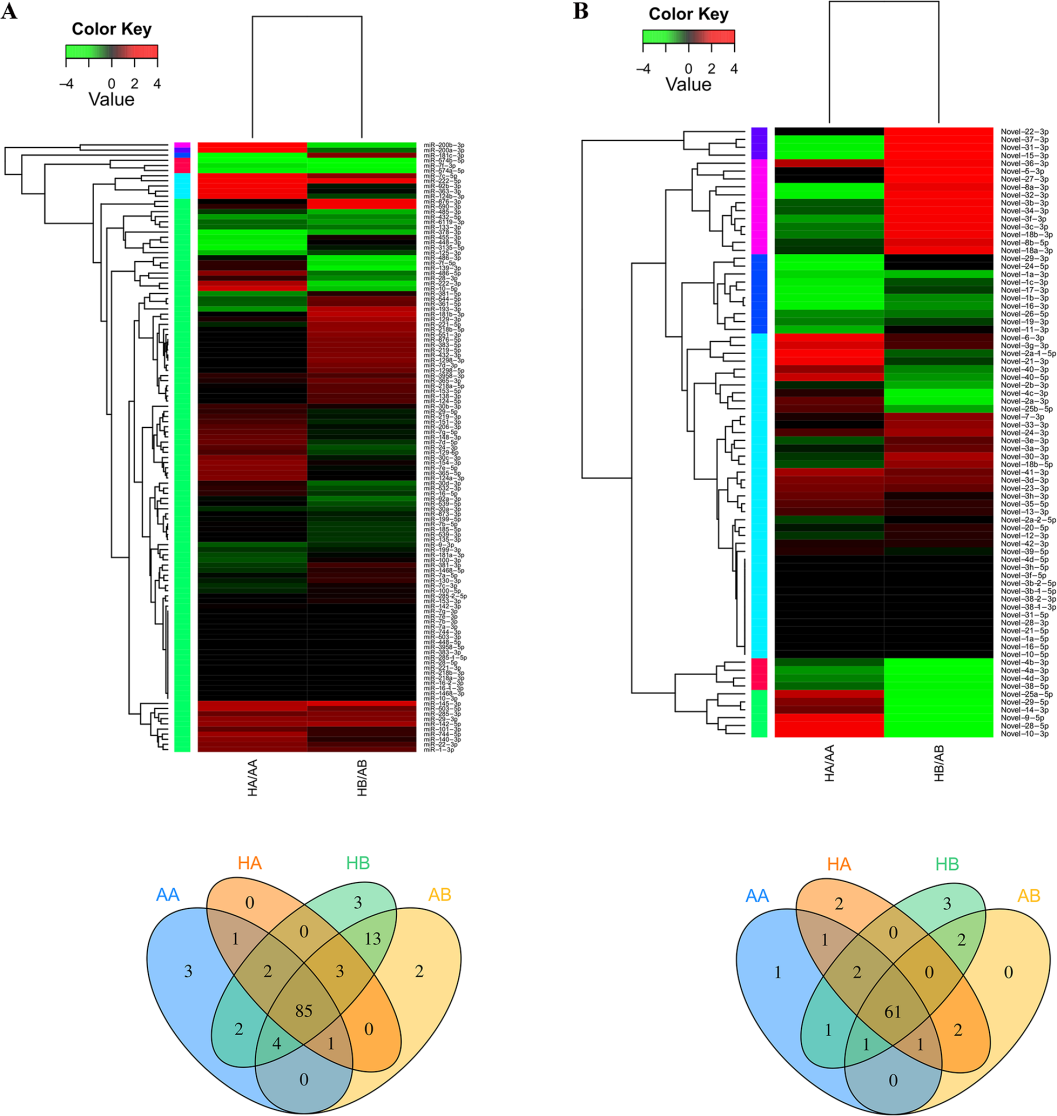
MiRNA expression patterns in four bat libraries. The expression of miRNAs identified in this study were calculated by sequence counts (Transcript Per Million, TPM). Heat maps represent the clustering of miRNAs (up) and the Venn diagram shows the number of miRNAs in each library (down). (A) 119 conserved miRNAs match to the Metazoan mature miRNAs in Sanger miRBase; (B) 77 Novel miRNAs identified by manual screening. Details of differentially expressed miRNAs are shown in S7 Table. HB: hibernating state brain; AB: active state brain; HA: hibernating state adipose tissue; AA: active state adipose tissue.

Finally, we obtained a total of 196 mature miRNAs, including 119 conserved miRNAs and 77 novel bat-specific miRNAs, and sequence raw counts varied from one to 1,946,308 (S7 Table). Further analysis showed that 12.2% (23/188, brain) and 4.6% (8/173, WAT) of the miRNA was expressed in a tissue-specific manner, whereas 10.1% (19/188, brain), and 10.9% (19/173, WAT) exhibited state-specific expression (Fig 3 and S7 Table). In the 31 state/tissue-specific miRNAs, six (miR-1298-5p, miR-124-5p, miR-153-5p, miR-153-3p, miR-551-3p and miR-1298-3p) had counts above 100, and they were all specifically expressed in the brain (S7 Table).

### Differential miRNA expression analysis and validation

A total 49 out of 196 miRNAs were differentially expressed (*P*≤0.01 & │logFC│≥1) between the hibernation vs. the active state, including 33 miRNAs in brain, 25 miRNAs in WAT and 9 miRNAs in both tissues (Table 2 and S7 Table). Of 33 miRNAs previously shown to be differentially expressed during torpor (S8 Table), ten (mir-1, mir-24, miR-29, miR-124a, mir-142, mir-181a, mir-181b mir-206, mir-378 and mir-486) were detected in our study (S7 Table). Moreover, we found that mir-378 was differentially expressed, and mir-142, mir-181b and mir-486 were also somewhat differentially expressed during hibernation (S7 Table).

**Table 2:** 49 differentially expressed miRNAs identified by Solexa sequencing in the brain or adipose tissues during hibernation.

		HA vs. AA				HB vs. AB			
Name	Total counts	logFC	logTPM	PValue	Mark	logFC	logTPM	PValue	Mark
miR-124b-3p	101576	2.259908382	5.6190503	0.0018537	UP	-0.536674	14.312553	0.4127694	DOWN
miR-139-3p	3183	0.37423932	8.5019562	0.5727299	UP	-2.1306716	8.4057206	0.0019162	DOWN
miR-200a-3p	334	7.578561186	3.2895048	2.45E-06	UP	-0.886215	5.923925	0.1986791	DOWN
miR-200b-3p	420	4.364567511	6.7026081	3.08E-08	UP	-1.8932045	4.6037305	0.0113473	DOWN
miR-222-3p	12491	1.429407306	10.720804	0.0321579	UP	-1.8021156	10.33994	0.0075425	DOWN
miR-222-5p	290	3.030812254	5.7697748	5.80E-05	UP	2.1583491	4.6655436	0.0044099	UP
miR-3135-5p	6682	-2.637365825	9.938779	0.0001553	DOWN	-0.1976903	8.1896251	0.7652108	DOWN
miR-363-3p	590	2.133071814	5.9211148	0.0028745	UP	-0.0815074	6.3009552	0.9118204	DOWN
miR-378-3p	198036	-2.818466705	15.121184	5.51E-05	DOWN	-1.5232279	10.232562	0.0228152	DOWN
miR-448-3p	8783	-2.143218357	7.3054357	0.0022535	DOWN	0.0288125	10.656037	0.9653039	UP
miR-455-3p	1010	-1.921311717	6.8853723	0.0061558	DOWN	0.1968478	6.3795769	0.7761823	UP
miR-486-3p	809	-0.156052775	6.9078207	0.825439	DOWN	-1.9680416	5.8518332	0.0056228	DOWN
miR-574a-5p	400	-2.521905714	6.1292436	0.0006325	DOWN	-2.7422469	2.7923067	0.0039648	DOWN
miR-574b-5p	153	-4.250113976	4.6476538	1.32E-05	DOWN	-3.8191272	1.987474	0.0030428	DOWN
miR-590-3p	136	0.378106825	4.4405947	0.6392554	UP	2.6462719	3.3179244	0.0035346	UP
miR-7c-5p	32436	2.712605457	8.7286431	0.0001116	UP	1.1902664	12.649161	0.0723922	UP
miR-7f-5p	227183	0.390233519	13.83588	0.5510089	UP	-1.8441761	15.019439	0.0062266	DOWN
miR-92b-3p	9041	2.884880248	5.7195942	0.0001081	UP	-0.1793825	10.807021	0.7843292	DOWN
Novel-10-3p	111	3.016403333	12.848138	0.0001838	UP	-4.4272759	12.275541	4.45E-06	DOWN
Novel-14-3p	97	0.936226836	12.677215	0.2187093	UP	-4.3606505	11.683673	3.70E-05	DOWN
Novel-15-3p	1669	-2.944335536	11.581472	0.0018222	DOWN	7.2036728	15.19745	3.14E-14	UP
Novel-16-3p	87	-2.344769395	12.036684	0.0062019	DOWN	-1.2012847	9.4737976	0.3901332	DOWN
Novel-17-3p	81	-2.304954477	12.263073	0.0052596	DOWN	-0.5052158	9.9593452	0.7250474	DOWN
Novel-18a-3p	1471	-0.426177519	15.116184	0.5299252	DOWN	2.3688352	16.00558	0.0006634	UP
Novel-18b-3p	344	-1.02311079	13.052043	0.1656782	DOWN	2.5486332	13.71619	0.0004685	UP
Novel-1b-3p	121	-2.293820099	12.911157	0.0026867	DOWN	-1.1555324	10.404784	0.2531532	DOWN
Novel-21-3p	1424	2.451014248	15.801168	0.0004914	UP	-0.4894437	15.830676	0.4628932	DOWN
Novel-22-3p	71	0	0	1	_	7.397751	11.114078	2.45E-06	UP
Novel-25a-5p	1088	1.581470973	15.986822	0.0200239	UP	-3.2647271	15.214248	8.67E-06	DOWN
Novel-27-3p	52	0	0	1	_	3.8813759	10.646132	0.0002438	UP
Novel-28-5p	50	2.607474245	11.636459	0.0034908	UP	-3.7091105	11.077865	0.0008149	DOWN
Novel-29-5p	50	1.097898289	11.646378	0.1811178	UP	-4.2594804	10.735381	0.0002438	DOWN
Novel-2a-3p	157	0.806179078	13.221511	0.2539667	UP	-1.9817143	12.411679	0.0100787	DOWN
Novel-2a-1-5p	38	3.353470354	10.81473	0.002812	UP	-0.7705951	10.858367	0.4453967	DOWN
Novel-31-3p	910	-2.058174551	12.059167	0.0145875	DOWN	5.722207	14.947979	2.83E-11	UP
Novel-32-3p	649	-2.308464878	13.31712	0.0020344	DOWN	3.1963163	13.574877	3.13E-05	UP
Novel-34-3p	329	-0.707605215	12.444825	0.3636482	DOWN	3.301607	13.981698	1.33E-05	UP
Novel-36-3p	309	1.484842174	11.704508	0.070754	UP	2.9220594	13.854956	8.30E-05	UP
Novel-37-3p	3841	-2.056735288	13.166302	0.0056955	DOWN	6.1069365	16.429287	4.25E-13	UP
Novel-38-5p	238	-0.844950584	13.898719	0.2282207	DOWN	-2.8099732	12.134971	0.0009641	DOWN
Novel-3b-3p	153	-0.732186096	11.093626	0.4453967	DOWN	2.9094233	12.254351	0.0004231	UP
Novel-3c-3p	127	-0.989181873	10.999282	0.2980811	DOWN	2.707911	11.810942	0.0012308	UP
Novel-3f-3p	66	-1.301092394	10.228842	0.2836528	DOWN	2.9447601	11.027109	0.0019476	UP
Novel-4a-3p	148	-1.243075915	13.337668	0.0862047	DOWN	-3.8778764	11.233557	0.0003435	DOWN
Novel-4b-3p	1962	-0.712484479	17.03775	0.2811898	DOWN	-4.5924403	15.198042	8.55E-09	DOWN
Novel-4d-3p	93	-1.074767845	12.588034	0.1491761	DOWN	-3.2491131	10.65837	0.0047291	DOWN
Novel-5-3p	146	0	0	1	_	4.8140123	11.573737	2.47E-06	UP
Novel-6-3p	139	2.426333279	12.19918	0.0032194	UP	0.5738677	12.756475	0.4367159	UP
Novel-9-5p	126	2.212281384	13.005661	0.0042355	UP	-5.0103113	12.29918	1.27E-06	DOWN

To validate miRNA expression level, we tested 10 differentially expressed miRNAs by miRNA-specific stem-loop qPCR. According to the results of Solexa sequencing, five of these miRNAs (mir-222-5p, mir-574a-5p, Novel-9-5p, Novel-10-3p and Novel-37-3p) were differentially expressed during hibernation in both the brain and WAT; another five miRNAs, including three (mir-7f-5p, mir-139-3p and mir-222-3p) differentially expressed only in the brain and two (mir-124b-3p and mir-378-3p) only in WAT, were used as negative controls. PCR confirmed the Solexa sequencing expression patterns (Table 3). In WAT (HA/AA), six miRNAs (mir-124b-3p, mir-222-5p, mir-378-3p, mir-574a-5p, Novel-9-5p and Novel-10-3p) showed consistent expression patterns and all reached significance by both technologies (Table 3). In the brain (HB/AB), the differentially expressed patterns of four miRNAs (mir-222-5p, Novel-9-5p, Novel-10-3p and Novel-37-3p) were identical in both technologies. Moreover, two negative controls, mir-7f-5p in WAT and mir-378-3p in brain showed no significant change detected in sequencing data, were confirmed by qPCR.

**Table 3:** Comparison of ten miRNA expression pattern in four libraries, as detected by Solexa sequencing and qPCR.

	HA vs. AA						HB vs. AB					
	Illumina			Real-time PCR			Illumina			Real-time PCR		
miRNA Name	logFC	*P*-value^a^	Mark	log2^−ΔΔCT^	*P*-value^b^	Mark	logFC	*P*-value^a^	Mark	log2^−ΔΔCT^	*P*-value^b^	Mark
miR-124b-3p	2.26	1.85E-03	↑ *	1.87	1.74E-03	↑ *	-0.542	0.41	↓	-0.98	0.12	↓
miR-139-3p	0.37	0.57	↑	-2.89	3.91E-04	↓ *	-2.13	1.92E-03	↓ *	-0.67	0.11	↓
miR-222-3p	1.43	0.03	↑	1.07	1.36E-05	↑ *	-1.8	7.54E-03	↓ *	-0.34	0.15	↓
miR-222-5p	3.03	5.79E-05	↑ *	0.31	6.81E-03	↑ *	2.16	4.41E-03	↑ *	0.59	7.67E-03	↑ *
miR-378-3p	-2.82	5.51E-05	↓ *	-0.82	1.57E-06	↓ *	-1.52	0.02	↓	-0.1	0.04	↓ *
miR-574a-5p	-2.52	6.32E-04	↓ *	-6.23	2.08E-07	↓ *	-2.74	3.96E-03	↓ *	-0.24	0.83	↓
miR-7f-5p	0.39	0.55	↑	-0.05	0.12	↓	-1.84	6.23E-03	↓ *	-0.796	0.24	↓
Novel-10-3p	3.02	1.84E-04	↑ *	1.05	0.01	↑ *	-4.43	4.45E-06	↓ *	-0.67	8.52E-07	↓ *
Novel-37-3p	-2.06	5.69E-03	↓ *	-0.04	0.25	↓	6.11	4.20E-13	↑ *	0.36	7.27E-03	↑ *
Novel-9-5p	2.21	4.24E-03	↑ *	35.07	1.69E-03	↑ *	-5.01	1.27E-06	↓ *	-1.86	3.82E-24	↓ *

PCR was performed in three biological replicates of each state with triplicate wells for each individual sample.

↑, up-regulated

↓, down-regulated

*, *P*-value^a^<0.01 or *P*-value^b^<0.05.

### Target prediction and annotation

To better understand the potential role and mechanism of miRNAs in hibernation, we predicted the miRNA target genes. Firstly, we obtained 22,432 *M*. *lucifugus* reference genes and 104,763 *H*. *sapiens* mRNAs and searched for orthologous genes by tblastx. A total of 20,282 *M*. *lucifugus* genes were orthologous to *H*. *sapiens* mRNAs with a BLAST e value ≤1e-10 and identity ≥60%. As 93.6% of human mRNAs have a 3’ UTR less than 3 Kb (S1 Fig), we extracted 3 Kb downstream of *M*. *lucifugus* coding sequences, including 2151 *M*. *lucifugus* genes with no human orthologs. Finally, we identified 87.8% (19,698 / 22,432) reference genes as the targets of 196 miRNAs. Both KEGG pathway and GO annotation analyses indicated that most physiological processes and cell functions are regulated by 196 miRNAs. We did not observe any significant differences in the number of target genes when we compared 119 conserved miRNAs vs. 196 miRNAs and 77 novel miRNAs vs. 196 miRNAs (S9 Table). In contrast, further analysis showed that many pathways in both tissues were markedly affected by the differentially expressed miRNAs, especially by the miRNAs down-regulated during hibernation (S9 Table).

**Fig 4:**
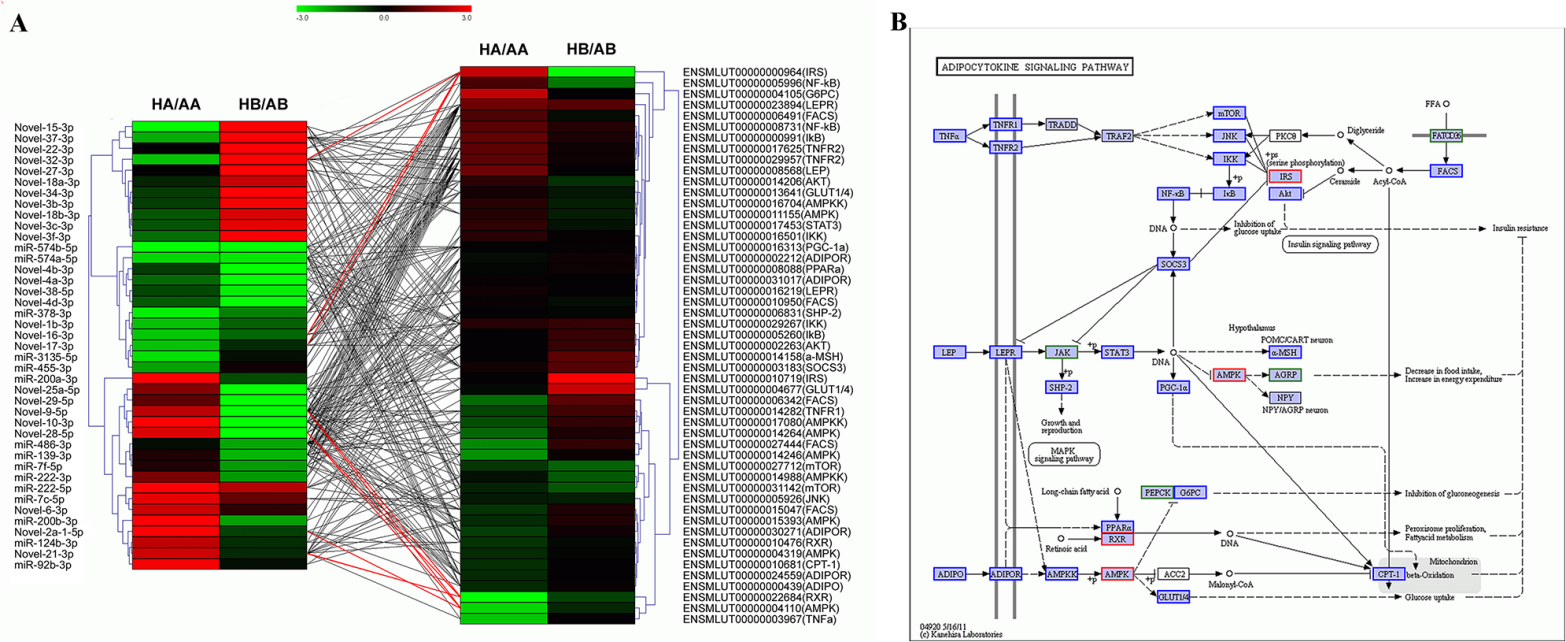
Co-regulation of miRNAs and target mRNAs in the adipocytokine signaling pathway. (A) Expression pattern and relationship of differentially expressed miRNAs (left) and mRNAs involved in the adipocytokine signaling pathway (right, identified by DGE, unpublished data). The expression profile of a miRNA or gene in the brain (HB/AB) and adipose tissue (HA/AA) was calculated by sequence counts (TPM). The red and green colors represent high and low gene expression after comparing the hibernating state vs. the active state. Differentially expressed miRNAs and mRNAs (*P*≤0.01) during hibernation are linked with red lines; (B) Network of adipocytokine signaling pathway. Red box, predicted target genes of differentially expressed miRNAs that were also identified by DGE; Blue box, predicted target genes of differentially expressed miRNAs, but that were not identified by DGE; Green box, genes identified by DGE.

We further examined the adipocytokine signaling pathway to determine the relationship between miRNAs and target mRNAs during hibernation, as adipocytokine signaling is co-regulated by both brain and adipose tissues. Moreover, we searched for mRNAs from our on-going project of digital gene expression (DGE) sequencing to enrich for target genes. DGE sequencing has been conducted on the same animals used here (unpublished data). A total of 64 mRNAs involved in adipocytokine signaling pathway were identified by DGE sequencing (S10 Table, DGE unpublished data). By combining the miRNA and target mRNA expression patterns, we found that 41 of 49 differentially expressed miRNAs regulated the expression of 51 genes, and more than half of the genes in each tissue were up-regulated during hibernation. Among these, the differential expression of three mRNAs (Insulin Receptor Substrate, IRS; Adenosine 5‘-monophosphate (AMP)-activated protein kinase, AMPK; Retinoid X receptor, RXR) was significant (*P*≤0.01), including IRS over-expression. The others were down-regulated during HA/AA (Fig 4 and S10 Table).

## Discussion

Our study shows that in the hibernating bat *Myotis ricketti*, 33 brain and 25 white adipose tissue (WAT) miRNAs were differentially expressed between euthermia and hibernation. Consequently, they are likely involved in regulating functional processes and energy metabolism during hibernation.

Our findings were possible with the ultrahigh-throughput sequencing technique (RNA-seq) Illumina/Solexa sequencing, which provides a great platform to discover and analyze small RNAs. It has been previously used in non-model species, such as the Arctic ground squirrel [*Urocitellus* (*Spermophilus) parryii*] and the sea cucumber (*Apostichopus japonicus*) during hypometabolism, but these species’ genomic backgrounds and miRNA data are not available in miRbase [18, 21]. The clear advantages of Solexa sequencing are the generation of several million small RNA sequences in each small RNA library of one run, the ability to identify novel miRNAs, the potential to overcome the drawbacks of microarrays, and the generation of expression profiles in a greater and reproducible dynamic range [53]. However, this technology can be influenced by sequencing errors and raw data processing before miRNA identification [53]. In the present study, we performed Solexa high-throughput sequencing with stem-loop qPCR validation of expression levels to analyze miRNAs involved in hibernation. Moreover, we chose a widely distributed bat species in China, congeneric with *Myotis lucifugus*, as the animal model to explore miRNA expression in two vital organs (brain and WAT) during hibernation.

Our data show that the sequencing information from four libraries was saturated, and 88.7% unique reads were tissue/state-specifically expressed (S3 Table). These data indicate a high quality of small RNA library construction and screening and that the libraries meet the needs of small RNA annotation and analysis. After mapping the unique reads to the *M*. *lucifugus* genome database and removing the reads that matched genome ≥20, the small RNA annotation indicated that miRNAs (47.7% in total) are the most abundant fraction of small RNAs (see Table 1 and Fig 1). These results are similar to those of previous miRNA profiling of multi-animal species [18, 21, 36]. Furthermore, we found that SINE and LINE, which are retroposons widely used as phylogenetic markers [54, 55], were the major source of genomic repeat-derived siRNAs in all four libraries. The detailed information of SINE and LINE provided us with the ability to further explore the phylogenetic evolution of heterothermy in mammals, especially in bats.

Our data also revealed a large number of conserved and novel miRNAs. We identified a total of 196 mature miRNAs, including 119 conserved miRNAs and 77 novel miRNAs (S7 Table). Moreover, we found that 31 (22 conserved and nine novel) of 196 miRNAs were expressed in a tissue/state-specific manner, suggesting a complex mechanism of miRNA regulation during hibernation. Of those, six conserved miRNAs with expression counts >100 were specifically expressed in the brain, including miR-153, which has been shown to function in neuronal protection and tumorigenesis [56, 57], miR-124, which regulates metabolic and immune processes [58, 59], miR-551, which is implicated in the stress response [60], and miR-1298, which has been associated with neural and endocrinological disease [61]. However, in the absence of direct and detailed evidence between the molecular data and animal function, it is necessary to further investigate the roles of these tissue- and state-specific miRNAs during hibernation.

Of 196 miRNAs, 49 were differentially expressed (*P*≤0.01 & │logFC│≥1) (Table 2 and S7 Table), including ten miRNAs that had been shown to regulate mammalian hibernation in other species (S8 Table) [18, 20, 22–25]. Both microarray and qPCR have been used to validate miRNA expression in many studies, as they are more accurate than RNA-seq sequencing [18, 21]. In our study, qPCR validation of 10 out of 49 differentially expressed miRNAs provided consistent results with those of Solexa sequencing (Table 3). We conclude that our data are reliable. Although we validated a few differentially expressed miRNAs by qPCR in our study, the limitations of RNA-seq, and the value of sample pooling in high-throughput sequencing, we suggest that microarray hybridization should be performed in the future to further validate the significance of differentially expressed miRNAs obtained here. Microarray analysis could potentially compensate for the drawbacks of RNA-seq sequencing [53] and estimate the biological variability.

To gain insight into the potential broader functions of the miRNAs we identified, we predicted their putative targets and classified them by KEGG pathway assay and GO annotation. Comparing the targets of 196 miRNAs, we found that miRNAs were differentially expressed in both brain and adipose tissues between euthermia and hibernation states, and this is likely a reflection of the different activities of many essential processes during these states (e.g., lipid metabolism and signal transduction, S9 Table). We found that only 49 of 196 miRNAs regulated most target gene expression (12,909 of 20,370), and thus most physiological processes during hibernation, contributing to reduced energy expenditure during hypometabolism. Moreover, both KEGG and GO assays identified the same enriched functional groups (*P*<0.05), and all of them were affected by differentially expressed miRNAs, particularly by down-regulated miRNAs (S9 Table). By binding to target mRNAs, miRNAs can reversibly degrade their targets at the transcriptional level and/or inhibit their translation [19]. Thus, during torpor, brain and WAT physiological processes were globally affected by down-regulated miRNAs, suggesting that these processes were activated, supporting our hypothesis. Moreover, previous studies have shown that some specific brain regions such as the hypothalamus are involved in regulating mammalian hibernation [62–64]. Our study was limited to the use of entire brains, but future work on specific brain regions may identify the precise regulation of miRNA expression in important brain areas. Such studies could further expand our knowledge of hibernation regulation.

The brain plays an important role in controlling and synchronizing peripheral physiology to reduce energy expenditure and minimizing tissue damage and/or disease during hibernation [3, 4, 7]. In contrast, adipose tissue is the primary energy source during hibernation and is especially critical during periodic rewarming [6, 65]. Thus, specific molecular changes are required to accommodate the specific roles of brain and adipose tissue during hibernation. Because the potential network of miRNAs and targets is highly complicated, we chose to examine the adipocytokine signaling pathway, which plays an important role in signaling transduction and energy metabolism [66, 67]. In both tissues, more than half mRNAs were up-regulated (Fig 4A) in contrast to miRNA expression, indicating that this pathway was activated to some extent. According to KEGG analysis, the adipocytokine signaling pathway can be subdivided into three interrelated parts, TNFα (Tumor Necrosis Factor α)-related genes, LEP (Leptin)-related genes and ADIPO (Adiponectin)-related genes (Fig 4B). Andrews et al. [13] previously showed that energy metabolism of hibernators is shifted at the gene level from glycometabolism in the liver and/or pancreas to lipid metabolism in adipose tissue during hibernation. We found that the significant up-regulation of IRS in WAT could be indicative of glucose uptake inhibition, whereas a decrease in AMPK in WAT could promote gluconeogenesis. Our data help to illustrate how miRNAs and mRNAs control the metabolic switch by inhibiting the glycometabolic pathway to burning fat as the main energy source, supporting previous reports [65]. While the regulation of fat metabolism is crucial during hibernation, the regulation of food intake is important during euthermia. Negative AMPK expression or the inhibition of AMPK and AMPK-related gene activity in the hypothalamus result in reduced food intake [68, 69]. We hypothesize that down-regulation of AMPK and RXR in WAT lead to decreased food intake and increased energy expenditure. Network construction increases our understanding of molecular mechanisms mediated by miRNAs and target genes. It also provides clues to uncover how brain and adipose tissue cooperate to regulate hibernation by decreasing food intake, inhibiting glucose metabolism and increasing fatty acid metabolism to meet the physiological needs of hibernators.

## Conclusions

We identified 196 miRNAs (77 novel miRNAs) in bats by in-depth analysis of the small RNAs in the hibernating species *Myotis ricketti*. Differential miRNA expression suggests that some physiological pathways in the brain and adipose tissue are activated during hibernation. In contrast to other peripheral tissues that are physiologically suppressed, our findings advance the understanding of the molecular mechanisms of hibernation. However, further study is required to validate the expression pattern during hibernation vs. euthermia, estimate biological variability, and elucidate the mechanisms of specific miRNAs and their target mRNAs in hibernation. Moreover, the Interspersed Elements (LINE and SINE) identified here may help further explore the phylogenetic evolution of heterothermy in bats. With the progress of the *M*. *lucifugus* genome sequencing project and the increased accuracy of gene annotation, more detailed information on miRNAs involved in hibernation will likely be uncovered from bat transcriptome libraries. These can be used to better understand heterothermy the physiology and evolution of heterothermy.

## Supporting Information

S1 Fig Analysis of 3’ UTR length of *Homo sapiens* mRNAs. *Homo sapiens* reference sequences were downloaded from the NCBI Reference Sequence database (http://www.ncbi.nlm.nih.gov/RefSeq/).
(JPG)

S2 Fig Flow chart of *Myotis ricketti* small RNA filtering and miRNA identification. (PNG)

S3 Fig Length distribution of known miRNA precursors of five organisms in metazoan miRBase. gga: *Gallus gallus*; has: *Homo sapiens*; cel: *Caenorhabditis elegans*; dme: *Drosophila melanogaster*; sme: *Schmidtea mediterranea*.
(JPG)

S1 Table Forward, stem-loop and universal primers used to amplify miRNAs and 5S rRNA. (DOC)

S2 Table Summary of Solexa small RNA reads statistics. The table summarizes the total number of small RNA reads by Solexa sequencing. After removing adapter contaminants, reads less than 18 nt and containing Ns, the clean reads from each library were used for the small RNA screen. HB: hibernating state brain; AB: active state brain; HA: hibernating state adipose tissue; AA: active state adipose tissue.
(DOC)

S3 Table Summary of small RNA read expression profiles in four libraries. Redundancy among libraries was calculated to estimate the quality of the Solexa small RNA libraries. Moreover, the numbers of state/tissue-specific expressed reads were identified. HB: hibernating state brain; AB: active state brain; HA: hibernating state adipose tissue; AA: active state adipose tissue.
(DOC)

S4 Table Summary of perfectly mapped total small RNA unique reads. (DOC)

S5 Table Summary statistics of unique miRNA read identification. (DOC)

S6 Table Details of miRNA hairpin screening. (A) 90 conserved miRNA hairpins. (B) 101 novel miRNA hairpins.
(XLSX)

S7 Table List of miRNA expression and distribution. (A) Expression details of 196 miRNAs (119 conserved miRNAs and 77 novel miRNAs). (B) List of 33 miRNAs differentially expressed in the brain (*P*≤0.01 & │logFC ≥1). (C) List of 25 miRNAs differentially expressed in adipose tissue (*P*≤0.01 & │logFC│≥1). (D) List of 9 miRNAs differentially expressed in both brain and adipose tissue (*P*≤0.01 & │logFC│≥1). (E) List of 31 tissue/state-specifically expressed miRNAs in brain (23) and adipose tissue (8).
(XLSX)

S8 Table Previously identified miRNAs involved in hibernation. (XLSX)

S9 Table Classification of miRNA target genes. KEGG pathway analysis (A) and GO annotation (B) of target genes regulated by 196 miRNAs, 119 conserved miRNAs, 8 conserved miRNAs differentially expressed in the brain, 13 conserved miRNAs differentially expressed in adipose tissue, 77 novel miRNAs, 25 novel miRNAs differentially expressed in the brain, and 12 novel miRNAs differentially expressed in adipose tissue. T-tests were performed to analyze the bio-activity of each physiological process in both tissues by comparing hibernation vs. active state.
(XLS)

S10 Table Summary of mRNAs identified by DGE sequencing. Genes, only identified by DGE sequencing and not the targets of differentially expressed miRNAs which obtained in this study, are in green.
(XLS)
