# Supplementary figures and images for: Down but Not Out: The Role of MicroRNAs in Hibernating Bats
Source: PLoS One. 2015 Aug 5;10(8):e0135064. doi: 10.1371/journal.pone.0135064 (PMC4526555; doi:10.1371/journal.pone.0135064)

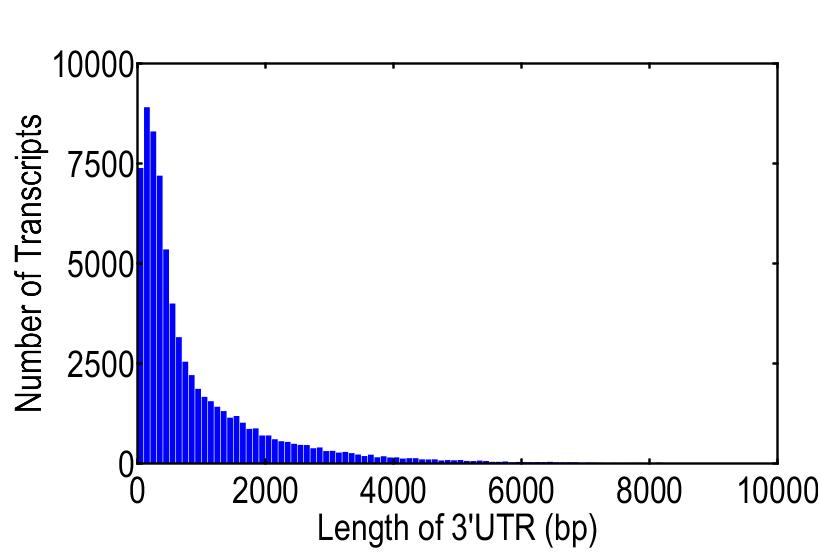

Supplement: S1 Fig — Homo sapiens reference sequences were downloaded from the NCBI Reference Sequence database (http://www.ncbi.nlm.nih.gov/RefSeq/). (JPG) [file pone.0135064.s001.JPG]

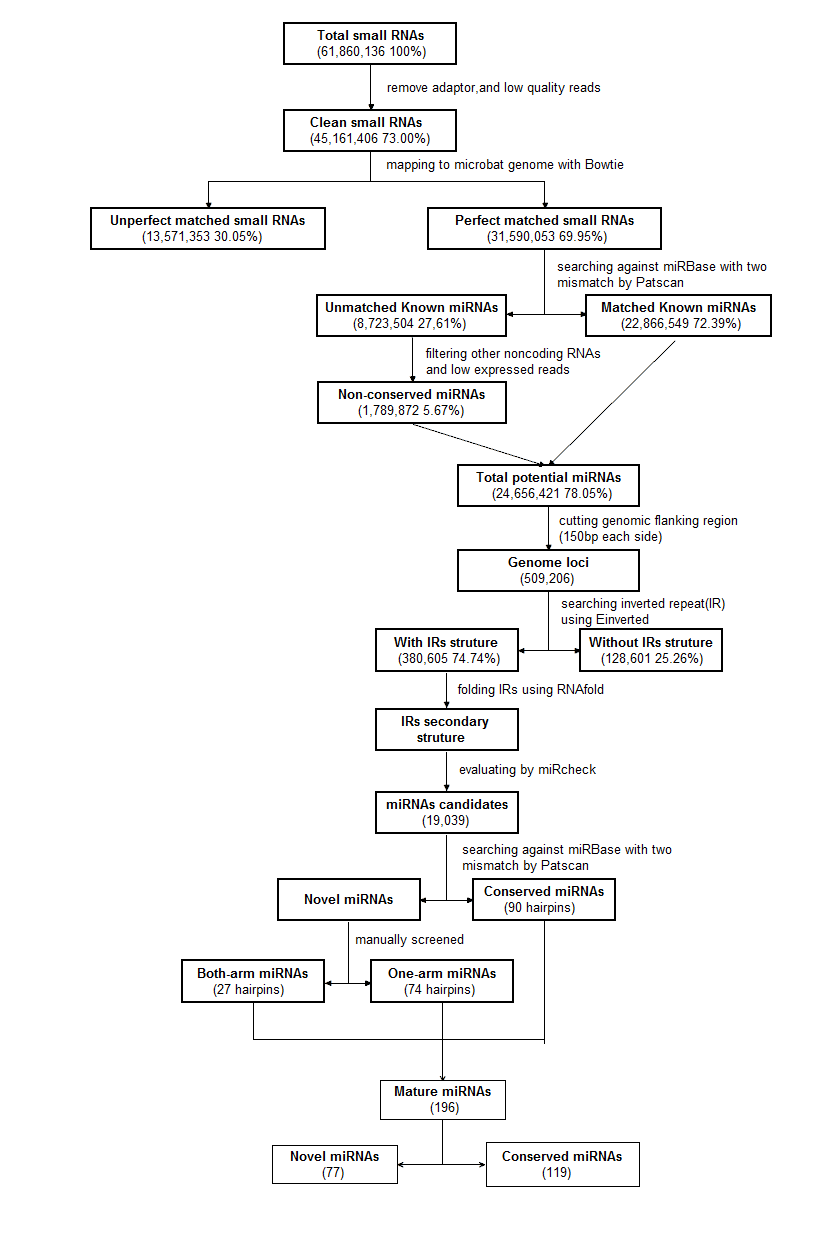

Supplement: S2 Fig — (PNG) [file pone.0135064.s002.png]

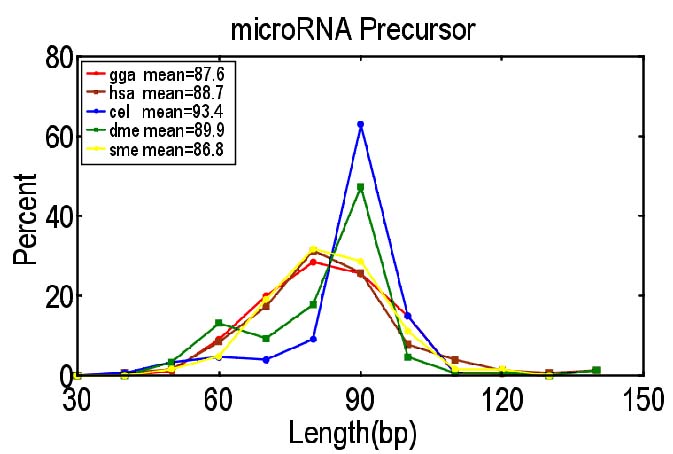

Supplement: S3 Fig — gga: Gallus gallus; has: Homo sapiens; cel: Caenorhabditis elegans; dme: Drosophila melanogaster; sme: Schmidtea mediterranea. (JPG) [file pone.0135064.s003.jpg]
