# Supplementary material for: Down but Not Out: The Role of MicroRNAs in Hibernating Bats
Source: PLoS One. 2015 Aug 5;10(8):e0135064. doi: 10.1371/journal.pone.0135064 (PMC4526555; doi:10.1371/journal.pone.0135064)
Supplement: S1 Table — (DOC) [file pone.0135064.s004.doc]

**Forward, stem-loop and universal primers used to amplify miRNAs and 5S rRNA.**

| **Primer** |  | **Sequence** |
| --- | --- | --- |
| **miR-124b** | **Fwd Stem** | 5’- ACACTCCAGCTGGGGTAAGGCACGCGGTGA-3’  5’- CTCAACTGGTGTCGTGGAGTCGGCAATTCAGTTGAGttggcat-3’ |
| **miR-139** | **Fwd Stem** | 5’- ACACTCCAGCTGGGGTGGAGACGCGGCCCTG-3’  5’- CTCAACTGGTGTCGTGGAGTCGGCAATTCAGTTGAGactccaa-3’ |
| **miR-222** | **Fwd Stem** | 5’- ACACTCCAGCTGGGGAGCTACATCTGGCTA-3’  5’- CTCAACTGGTGTCGTGGAGTCGGCAATTCAGTTGAGgacccag-3’ |
| **miR-222*** | **Fwd Stem** | 5’- ACACTCCAGCTGGGGGGCTCAGTAGT-3’  5’- CTCAACTGGTGTCGTGGAGTCGGCAATTCAGTTGAGtacactg-3’ |
| **miR-378** | **Fwd Stem** | 5’- ACACTCCAGCTGGGGACTGGACTTGGAGTC-3’  5’- CTCAACTGGTGTCGTGGAGTCGGCAATTCAGTTGAGgccttct-3’ |
| **miR-574a** | **Fwd Stem** | 5’- ACACTCCAGCTGGGGTGAGTGTGTGTG-3’  5’- CTCAACTGGTGTCGTGGAGTCGGCAATTCAGTTGAGactcaca-3’ |
| **miR-7f** | **Fwd Stem** | 5’- ACACTCCAGCTGGGGAGAGGTAGTAGGTTG-3’  5’- CTCAACTGGTGTCGTGGAGTCGGCAATTCAGTTGAGaactctg-3’ |
| **Novel-10** | **Fwd Stem** | 5’- ACACTCCAGCTGGGGGGGGCACGCCGGC-3’  5’- CTCAACTGGTGTCGTGGAGTCGGCAATTCAGTTGAGccgcaag-3’ |
| **Novel-37** | **Fwd Stem** | 5’- ACACTCCAGCTGGGGTAGGAGCAGTGATAG-3’  5’- CTCAACTGGTGTCGTGGAGTCGGCAATTCAGTTGAGatattac-3’ |
| **Novel-9** | **Fwd Stem** | 5’- ACACTCCAGCTGGGGAGGAGGGAGCTGA-3’  5’- CTCAACTGGTGTCGTGGAGTCGGCAATTCAGTTGAGacatcga-3’ |
| **Universal** | **Rev** | 5’- TGGTGTCGTGGAGTCG-3’ |
| **5S rRNA** | **Fwd Stem** | 5’- ACGGCCATACAACCTTGAAC-3’  5’- GGTATTCCCAGGCGGTCT-3’ |
