# Supplementary material for: Down but Not Out: The Role of MicroRNAs in Hibernating Bats
Source: PLoS One. 2015 Aug 5;10(8):e0135064. doi: 10.1371/journal.pone.0135064 (PMC4526555; doi:10.1371/journal.pone.0135064)
Supplement: S2 Table — The table summarizes the total number of small RNA reads by Solexa sequencing. After removing adapter contaminants, reads less than 18 nt and containing Ns, the clean reads from each library were used for the small RNA screen. HB: hibernating state brain; AB: active state brain; HA: hibernating state adipose tissue; AA: active state adipose tissue. (DOC) [file pone.0135064.s005.doc]

**Summary of Solexa small RNA reads statistics.**

The table summarizes the total number of small RNA reads by Solexa sequencing. After removing adapter contaminants, reads less than 18 nt and containing Ns, the clean reads from each library were used for the small RNA screen. HB: hibernating state brain; AB: active state brain; HA: hibernating state adipose tissue; AA: active state adipose tissue.

|  | HB | | AB | | HA | | AA | |
| --- | --- | --- | --- | --- | --- | --- | --- | --- |
|  | Counts | Percentage | Counts | Percentage | Counts | Percentage | Counts | Percentage |
| Total Reads | 15640606 | 100.0 | 15435341 | 100.0 | 15292589 | 100.0 | 15491600 | 100.0 |
| Low Quality | 1504630 | 9.62 | 4288968 | 27.79 | 2238918 | 14.64 | 2667703 | 17.22 |
| High Quality | 14135976 | 90.38 | 11146373 | 72.21 | 13053671 | 85.36 | 12823897 | 82.78 |
| adaptor3’ | 3274 | 0.02 | 29877 | 0.19 | 15879 | 0.1 | 8203 | 0.05 |
| insert | 43384 | 0.28 | 37384 | 0.24 | 83211 | 0.54 | 80544 | 0.52 |
| 5' adaptor contaminants | 548961 | 3.51 | 409321 | 2.65 | 680547 | 4.45 | 612912 | 3.96 |
| size < 18 bp | 1378455 | 8.81 | 656854 | 4.26 | 723739 | 4.73 | 683641 | 4.41 |
| polyA | 1797 | 0.01 | 49 | 0 | 336 | 0 | 143 | 0 |
| size ≥ 18 bp | 12160105 | 77.75 | 10012888 | 64.87 | 11549959 | 75.53 | 11438454 | 73.84 |
