# Supplementary material for: Down but Not Out: The Role of MicroRNAs in Hibernating Bats
Source: PLoS One. 2015 Aug 5;10(8):e0135064. doi: 10.1371/journal.pone.0135064 (PMC4526555; doi:10.1371/journal.pone.0135064)
Supplement: S3 Table — Redundancy among libraries was calculated to estimate the quality of the Solexa small RNA libraries. Moreover, the numbers of state/tissue-specific expressed reads were identified. HB: hibernating state brain; AB: active state brain; HA: hibernating state adipose tissue; AA: active state adipose tissue. (DOC) [file pone.0135064.s006.doc]

**Summary of small RNA read expression profiles in four libraries.**

Redundancy among libraries was calculated to estimate the quality of the Solexa small RNA libraries. Moreover, the numbers of state/tissue-specific expressed reads were identified. HB: hibernating state brain; AB: active state brain; HA: hibernating state adipose tissue; AA: active state adipose tissue.

| Class | Total Reads | High-quality Reads | Total Clean  Unique Reads | Redundancy ^a^ / Percentage ^b^ (%) | Perfect Matched  Genome ^c^ | Percentage  (%) | Total of Perfect Matched | Percentage  (%) |
| --- | --- | --- | --- | --- | --- | --- | --- | --- |
| HB | 15640606 | 12160105 | 629751 | 94.82 ^a^ | 201875 | 32.06 | 8682951 | 71.41 |
| AB | 15435341 | 10012888 | 415452 | 95.85 ^a^ | 120695 | 29.05 | 7266543 | 72.57 |
| HA | 15292589 | 11549959 | 1164049 | 89.92 ^a^ | 373224 | 32.06 | 7502137 | 64.95 |
| AA | 15491600 | 11438454 | 536995 | 95.31 ^a^ | 152674 | 28.43 | 8138422 | 71.15 |
| Total | 61860136 | 45161406 | 2323170 | 94.86 ^a^ | 720862 | 31.03 | 31590053 | 69.95 |
| Co-expressed in four libraries |  | 40476153 | 53489 | 2.3 ^b^ | 22203 | 3.0 | 30250515 |  |
| Co-expressed in three libraries |  | 892666 | 53674 | 2.3 ^b^ | 13288 | 1.8 | 261944 |  |
| HB & AB & AA |  | 199462 | 9720 |  | 1965 |  | 70373 |  |
| AB & HA & AA |  | 178235 | 12651 |  | 2079 |  | 30059 |  |
| HB & AB & HA |  | 221411 | 12175 |  | 3879 |  | 69558 |  |
| HB & HA & AA |  | 293558 | 19128 |  | 5365 |  | 91954 |  |
| Co-expressed in two libraries |  | 961936 | 155262 | 6.6 ^b^ | 34421 | 4.8 | 198311 |  |
| AB & AA |  | 40808 | 11705 |  | 2265 |  | 7746 |  |
| HB & AB |  | 337147 | 32881 |  | 5039 |  | 55347 |  |
| AB & HA |  | 54195 | 14884 |  | 3789 |  | 14657 |  |
| HB & AA |  | 47546 | 14938 |  | 3197 |  | 11101 |  |
| HA & AA |  | 336523 | 51317 |  | 11285 |  | 66876 |  |
| HB & HA |  | 145717 | 29537 |  | 8846 |  | 42584 |  |
| Specific expressed in one libraries |  | 2830651 | 2060745 | 88.7 ^b^ | 650950 | 90.3 | 879283 |  |
| HB |  | 543022 | 457883 |  | 151381 |  | 181888 |  |
| AB |  | 305552 | 267947 |  | 79476 |  | 90603 |  |
| HA |  | 1501554 | 970868 |  | 315778 |  | 462484 |  |
| AA |  | 480523 | 364047 |  | 104315 |  | 144308 |  |

^a^ Redundancy(%)=(1 - Total Clean Unique Reads / High-quality Reads)*100%

^b^ Percentage(%)=Co-expressed Reads or Specific expressed Reads / Total Clean Unique Reads *100%

^c^ using Microbat (*Myotis lucifugus*) genome v71 downloaded from <http://www.ensembl.org/index.html> and bowtie 0.12.8 aligner: -v 0 -a
