# Supplementary material for: Down but Not Out: The Role of MicroRNAs in Hibernating Bats
Source: PLoS One. 2015 Aug 5;10(8):e0135064. doi: 10.1371/journal.pone.0135064 (PMC4526555; doi:10.1371/journal.pone.0135064)
Supplement: S4 Table — (DOC) [file pone.0135064.s007.doc]

**Summary of perfectly mapped total small RNA unique reads.**

| Matced Times | Total Unique Reads | |
| --- | --- | --- |
|  | Count | Percentage |
| 1 | 534555 | 74.15 |
| 2 | 65544 | 9.09 |
| 3 | 25117 | 3.48 |
| 4 | 15552 | 2.16 |
| 5 | 12211 | 1.69 |
| 6 | 7815 | 1.08 |
| 7 | 5297 | 0.73 |
| 8 | 3916 | 0.54 |
| 9 | 2985 | 0.41 |
| 10 | 2338 | 0.32 |
| 11 | 2102 | 0.29 |
| 12 | 1634 | 0.23 |
| 13 | 1463 | 0.20 |
| 14 | 1329 | 0.18 |
| 15 | 1126 | 0.16 |
| 16 | 875 | 0.12 |
| 17 | 801 | 0.11 |
| 18 | 679 | 0.09 |
| 19 | 562 | 0.08 |
| 20 | 567 | 0.08 |
| >20 | 34394 | 4.77 |
