# Supplementary material for: Down but Not Out: The Role of MicroRNAs in Hibernating Bats
Source: PLoS One. 2015 Aug 5;10(8):e0135064. doi: 10.1371/journal.pone.0135064 (PMC4526555; doi:10.1371/journal.pone.0135064)
Supplement: S5 Table — (DOC) [file pone.0135064.s008.doc]

**Summary statistics of unique miRNA read identification.**

| Class | Total | | | |
| --- | --- | --- | --- | --- |
|  | Number of unique reads | Percentage | Total sequences | Percentage |
|  |  |  |  |  |
| Total Distinct Reads | 2323170 | 100.00 | 45161406 | 100.00 |
| Total Perfect Matched | 720862 | 31.03 | 31590053 | 69.95 |
| Conserved In Metazoa^a^ | 6950 | 0.96 | 22866549 | 72.39 |
| Non-Conserved In Metazoa | 713912 | 99.04 | 8723504 | 27.61 |
| Total Filter | 561055 | 77.83 | 6933632 | 21.95 |
| Matched ncRNA (except microRNA)^b^ | 71131 | 9.87 | 6443708 | 20.40 |
| rRNA | 39273 | 5.45 | 5495818 | 17.40 |
| tRNA | 18189 | 2.52 | 244828 | 0.78 |
| snoRNA | 2360 | 0.33 | 36480 | 0.12 |
| snRNA | 454 | 0.06 | 7249 | 0.02 |
| other Nc-RNA | 10855 | 1.51 | 659333 | 2.09 |
| Low Expressed (Sequenced Times = 1) | 489924 | 67.96 | 489924 | 1.55 |
| Total Remain | 152857 | 21.20 | 1789872 | 5.67 |
| Total Potential Reads^c^ | 159807 | 22.17 | 24656421 | 78.05 |

^a^ Using Patscan with default parameters and two mismatches allowed to identify homologs of known metazoa miRNAs.

^b^ RNAs annotation of UCSC and Sanger Rfam database release 10.0 ,except microRNA

^c^ Total Potential Unique=Conserved In Metazoa + Total Remained In Non-Conserved
